# Supplementary material for: Observing COVID-19 related behaviors in a high visitor use area of Arches National Park
Source: PLoS One. 2021 Feb 22;16(2):e0247315. doi: 10.1371/journal.pone.0247315 (PMC7899324; doi:10.1371/journal.pone.0247315)
Supplement: Attachment — Submitted filename: Review Comments.docx [file pone.0247315.s001.docx]

| Comment | Resolution |
| --- | --- |
| 1. Please ensure that your manuscript meets PLOS ONE's style requirements, including those for file naming. The PLOS ONE style templates can be found at  <https://journals.plos.org/plosone/s/file?id=wjVg/PLOSOne_formatting_sample_main_body.pdf> and  <https://journals.plos.org/plosone/s/file?id=ba62/PLOSOne_formatting_sample_title_authors_affiliations.pdf> | Done – we have revamped citations and references and checked main body formatting. |
| 2. We note that you have indicated that data from this study are available upon request. PLOS only allows data to be available upon request if there are legal or ethical restrictions on sharing data publicly. For more information on unacceptable data access restrictions, please see <http://journals.plos.org/plosone/s/data-availability#loc-unacceptable-data-access-restrictions>.  a) If there are ethical or legal restrictions on sharing a de-identified data set, please explain them in detail (e.g., data contain potentially sensitive information, data are owned by a third-party organization, etc.) and who has imposed them (e.g., an ethics committee). Please also provide contact information for a data access committee, ethics committee, or other institutional body to which data requests may be sent.  b) If there are no restrictions, please upload the minimal anonymized data set necessary to replicate your study findings as either Supporting Information files or to a stable, public repository and provide us with the relevant URLs, DOIs, or accession numbers. For a list of acceptable repositories, please see <http://journals.plos.org/plosone/s/data-availability#loc-recommended-repositories>. | We are not able to provide images and videos as an accessible data source. This would violate the consent and privacy of individuals on the study.  We have provided the data set that was created from observing the video in a data repository. This is the data set that was ultimately used for the analysis. It is linked at our university’s repository:  <https://doi.org/10.26078/ymtv-2w67> |
| 3. We note that Figure 1 includes an image of a participant in the study. | This image is replaced. No participants are displayed in any figures. |
| - Please provide SI units throughout the paper. | We have placed SI units all throughout the paper where appropriate. |
| Line 69: I suggest adding ”entire” before April (to distinguish that is is not part of March and April) | We have corrected this sentence as suggested. It now reads:  “…closing of ARCH for a portion of March, the entirety of April, and most of May of 2020” |
| Paragraph starting with Line 83: please provide some references for CDC guidelines and the statements about ”behaviour” of NPS | Thank you for this comment. We have now cited a CDC website that details how people should behave when recreating in parks.  https://www.cdc.gov/coronavirus/2019-ncov/daily-life-coping/visitors.html |
| Sentence starting in line 100: It makes me wonder whether the Authors are in any capacity connected to the observed site (not necessarily in sense of conflict of interest, more so to gain insight into why you chose this particular site) | We chose this site in consultation with park managers as stated in the manuscript. We relied on their expertise and concerns about COVID-19. We feel this is accurately reflected in the manuscript as written. |
| Sampling details (line 125 onward): Please discuss how the chosen dates are representative of the general (pre-COVID) visitation patterns, and how representative it is to the camera downtime (or do the numbers of visitors drop so significantly that it does not warrant observation?) | The visitation conditions on these days were slightly lower than but generally consistent with July visitation in previous years. In essence, visitation patters had returned to normal after the COVID 19 induced shutdown and during the hours of our sampling, the park experienced a typical “morning rush”.  We have updated the manuscript to now state:  “The extreme temperatures also impacted the camera operation by interfering with the motion detection, so the sample of video images was restricted to when the camera operated properly yielding images on July 12 from 7:17 AM to 11:10 AM and on July 14 from 7:18 AM to 7:58 AM, 10:04 AM to 10:54 AM, and 11:24 AM to 11:47 AM (see Figure 3). As shown in Figure 2, we have captured the busy morning period when encounters would seem to be most likely.” |
| Recording visitor behaviors (line 140 onward): It is not clear whether there was a single coder for the videos. How did you control for the quality of coding? | We now stated:  “One person was responsible for the majority of the coding, and other members of the research team checked their coding in randomly selected clips to ensure reliability.” |
| Line 152: I wonder whether there was an instance where a person was facing away from the camera and it was thus impossible to determine whether they are wearing a mask. How did you code the data in such case (if applicable)? | Excellent comment. We have added the following sentence to clarify:  “If an individual was facing away from the camera for the entire recording or was seen with a face covering not worn properly over the nose and mouth for the entire recording, they would not be counted as wearing a face covering.” |
| Line 155: Please add a brief discussion on how reliable is the use of reference points to determine distance and/or how it might impact the results if there could be such impact. | Our group visited the site and measured out distances between reference points. We have edited this paragraph to both clarify this point and provide a statement of limitations:  “For instance, we assumed adult height to be approximately 5-6 feet (1.52-1.83 meters) and used this to estimate interaction distances. If any individual in a group interacted with any individual from another group at a distance estimated to be <6 feet (1.83 meters), it was recorded as an interaction for the entire group. Other reference points, such as the length of the colored sidewalk portions, were measured on site. The use of reference points was necessary due to the 2-dimensional nature of the videos. There may, however, be an unknown amount of error related to the use of these reference points during observations. To counter some of this unknown error, the data recorder could classify the encounter as definite or probable; less than ten percent of the encounters fell into the probable category. We used the more conservative definition of an encounter and did not include probable encounters in the analysis. “ |
| Line 211: I suggest removing the word ”only” as we should avoid evaluating the results before discussion. | We have removed “only” from the beginning of this sentence. |
| Explaining the probability of encounters (line 217 onward): Please elaborate/provide more detail (I feel this part is significant, as you go beyond ”counting” and provide novel insights). What is the practical significance of the model results (e.g., pseudo r2)? | Rather than using the pseudo R-square, we provide actual and predicted probabilities that includes a new section of text and a new table (see table 3 in manuscript and text below):  “Model 2 does a good job of modeling the probabilities of encounters as shown in Table 3 where the actual and predicted probabilities from the sample show a close fit. From a practical perspective it is more useful to examine how varying group size and the number of groups impacts the probability of an encounter (see Figure 9 below).”  We also address the practical significance of this through explicitly pointing it out in Figure 9.  We have also added the following text to further emphasize this:  “Managing COVID-19 exposure is about individual risk management. The encounters we observed were brief and outdoors which mitigates the risk. However, these were observations lasting only one minute, individuals have the potential for many interactions over the visit and ARCH sees over 1.5 million visitors a year, so small changes in probabilities have the potential for large effects” |
| Line 221: Did you mean Table 2? | Yes – thank you for catching this. We have corrected this |
| Line 240: This statement could also be provided somehow in the introduction (as a research aim) | We have dialed this statement back a bit to have it align better with our original intention. Although the crux of this paper is about the predictive model that explains visitor social distancing behaviors, we also explicitly state that we will also use descriptive statistics to help understand visitor compliance with CDC guidelines. In this way, we now match the discussion to our initially stated research purpose of using descriptive statistics to further understanding COVID-19 risk behaviors. The paragraph now leads:  “Part of this work was to provide basic descriptive information about visitor compliance with CDC guidelines. We found over 60% of the visitors observed chose wore a face covering. This appears to conform with the health department’s guidance asking people to wear face coverings outdoors “when social distancing of 6 feet [1.83 meters] is not possible, reasonable, or prudent.” Visitors also managed their behavior in ways that place fewer people in proximity to one another when they are approaching facilities…” |
| Please elaborate the discussion by adding some critical evaluation of the results and their use. You state in the first sentence of the Discussion that you wanted to determine whether visitors comply with CDC guidelines, but then you only provide the percentage of visitors wearing mask without discussing the actual finding. The other percentages also seem moderate, and some statements in the first paragraph of page 12 seem a bit ambitious. You provide some recommendations and discuss the possibility of generalization of the findings, but I was missing something beyond describing the results. Were your research questions answered? How are the results useful for this and/or other parks? | We have rephrased that first sentence to focus on more descriptive aspects than determinative ones. To date and to the authors’ knowledge, no one has done this and it provides important context to the COVID-19 pandemic.  We also match some of these descriptive elements to the results from our model.  We have also made substantial additions to the first paragraph to connect our discussion to the research question. It now reads:  “Ultimately, the purpose of this research was to determine if visitors are able to socially distance in a crowded national park. Using observations near the visitor center at Arches National Park, we found evidence through our modeling that most visitors (68.8%) are able to avoid encounters with other groups. However, increasing the number of groups and the number of people in groups both drove increased risk of encounters. In sum, the large majority of visitors are able to navigate these crowded spaces in a way that reduces their risk of encounters. Part of the reason for this is likely due to the fact that so many people are traveling as a single-person group. For example, in our sample over 50% of the visitors were in a single person group. Research in the summer of 2016, demonstrated that only 7% of the visitation to Arches national Park were in a group size of one (RSG Environmental Consulting, 2017). Although it is possible more people are traveling alone, it is also conceivable that visitors are either taking turns to approach the facility or only one member of the group is choosing to visit the visitor center, enabling the rest of the party to remain in the parking lot.”  We have also more explicitly addressed some of the limitations and implications of this work through the following additions:  “However, we also found that face coverings were not predictive of encounters with other groups. In a real sense, this means that groups not wearing face coverings are no more or less likely to have encounters with other groups. Thus, the roughly 40% of visitor who do not wear masks do pose some risk of disease transmission through <6 foot (1.83 meter) encounters with other groups.”  “Although it is possible more people are traveling alone, it is also conceivable that visitors are either taking turns to approach the facility or only one member of the group is choosing to visit the visitor center, enabling the rest of the party to remain in the parking lot.” |
| Sentence starting in line 248: How could you determine that from your observations? Could we also stipulate that the person came alone (and that there is a rise of one-person groups now)? | We have clarified this with the caveat stated by the reviewer:  “Although it is possible more people are traveling alone, it is also conceivable that visitors are either taking turns to approach the facility or only one member of the group is choosing to visit the visitor center, enabling the rest of the party to remain in the parking lot.” |
| Line 254: How do you interpret this percentage? Is that enough/a lot/not enough? Please elaborate your discussion. | As scientists working in an applied field with managers, this is a qualitative call that we do not feel will add meaningfully to the discussion. Ultimately, park managers need to decide if this is sufficient for their management needs.  For example, in our sample over 50% of the visitors were in a single-person group which is substantially larger than recorded in research in the summer of 2016 which demonstrated that only 7% of the visitation to Arches National Park were in a group size of one (RSG Environmental Consulting, 2017). We touch on this in the comment immediately above this one. |
| Line 271: Seems a bit ambitious. | We softened the language in this sentence by adding the word “generally.” We feel that this sentence accurately reflects the findings in the manuscript. |
| Line 273: Consider replacing ”notoriously” (this was not the impression I got from the paper, and I personally never heard of the park before so all the information I have about the park were provided by you). | We have removed the word “notoriously” from the sentence. |
| Sentence starting in line 274: Based on your observations, you cannot make draw causal conclusions. I suggest rewording this part of the paragraph. | We have deleted this speculative sentence. |
| Line 278: How do your recommendations differ from the current recommendations to the visitors? Are they viable in practice? | Thank you for this comment. As this is an ever-evolving and new situation we all find ourselves in, it is a bit difficult to determine “current.” The parks we know of – including the study site – are currently providing the previously mentioned CDC guidelines to visitors through signage and other information. Adding this additional recommendation – small group travel when possible – seems both viable and reasonable giving the context. But ultimately, managers of those parks need to determine whether/when/where these recommendations are appropriate for their settings. |
| Figure 2: Please provide time-span for the plotted data | We have added this. Thank you. |
| Figure 8: Consider using the full word ”group” | We have changed this to “group.” This definitely makes things clearer. |
| Figure 9: Please provide the label for y-axis | We corrected this accidental omission. Thank you. |
